# Role of Myosin Va in the Plasticity of the Vertebrate Neuromuscular Junction *In Vivo*

**DOI:** 10.1371/journal.pone.0003871

**Published:** 2008-12-05

**Authors:** Ira Verena Röder, Yvonne Petersen, Kyeong Rok Choi, Veit Witzemann, John A. Hammer, Rüdiger Rudolf

**Affiliations:** 1 Institute of Toxicology and Genetics, Research Center Karlsruhe, Eggenstein-Leopoldshafen, Germany; 2 Max-Planck-Institute for Medical Research, Heidelberg, Germany; 3 Laboratory of Cell Biology, National Institutes of Health, Bethesda, Maryland, United States of America; Baylor College of Medicine, United States of America

## Abstract

**Background:**

Myosin Va is a motor protein involved in vesicular transport and its absence leads to movement disorders in humans (Griscelli and Elejalde syndromes) and rodents (e.g. *dilute lethal* phenotype in mice). We examined the role of myosin Va in the postsynaptic plasticity of the vertebrate neuromuscular junction (NMJ).

**Methodology/Principal Findings:**

*Dilute lethal* mice showed a good correlation between the propensity for seizures, and fragmentation and size reduction of NMJs. In an aneural C2C12 myoblast cell culture, expression of a dominant-negative fragment of myosin Va led to the accumulation of punctate structures containing the NMJ marker protein, rapsyn-GFP, in perinuclear clusters. In mouse hindlimb muscle, endogenous myosin Va co-precipitated with surface-exposed or internalised acetylcholine receptors and was markedly enriched in close proximity to the NMJ upon immunofluorescence. *In vivo* microscopy of exogenous full length myosin Va as well as a cargo-binding fragment of myosin Va showed localisation to the NMJ in wildtype mouse muscles. Furthermore, local interference with myosin Va function in live wildtype mouse muscles led to fragmentation and size reduction of NMJs, exclusion of rapsyn-GFP from NMJs, reduced persistence of acetylcholine receptors in NMJs and an increased amount of punctate structures bearing internalised NMJ proteins.

**Conclusions/Significance:**

In summary, our data show a crucial role of myosin Va for the plasticity of live vertebrate neuromuscular junctions and suggest its involvement in the recycling of internalised acetylcholine receptors back to the postsynaptic membrane.

## Introduction

Vertebrate neuromuscular junctions (NMJs) are the synapses between motoneurones and skeletal muscle fibres and mediate any kind of voluntary movement [for review, 1],[2]. The postsynaptic face of NMJs is rich in nicotinic acetylcholine receptors (AChRs) and other specific proteins, such as the AChR clustering factor rapsyn [3]–[5]. NMJs form during embryogenesis and are maintained after a perinatal period of synapse rearrangements [6], [7] in an essentially stable manner for long time periods [8], [9], presumably for the entire life span of a muscle fibre. Despite this long persistence of the overall structure, individual NMJ components such as AChRs have much shorter life spans, usually in the range of days [10]. The analysis of AChR degradation led to the identification of two metabolically distinct AChR populations [11], [12], so-called junctional (or endplate) and extrajunctional AChRs. While junctional AChRs were found to have a halflife of about 10 days, extrajunctional AChRs appear to decrease in number in the first two postnatal weeks and to exhibit a halflife of only about 1 day [13]. Also, the structural and functional properties of AChRs change during early postnatal development, since embryonic-type AChRs with an alpha(2)-beta-gamma-delta subunit composition are replaced by adult AChRs consisting of alpha(2)-beta-epsilon-delta subunits [14]. How and whether the differences in function and molecular composition of AChRs could be related to AChR degradation is not well understood. However, factors that are known to affect AChR stability are innervation and muscle activity [15]–[17].

In Torpedo electrocytes, AChRs were shown to be co-transported with rapsyn in vesicular carriers [18], and in heterologous tissue culture cells, a rapsyn-GFP fusion protein was found to travel along the cytoskeleton from the Golgi apparatus towards the cell surface [19], [20]. Given the large discrepancy between the lifetimes of NMJs and AChRs, there is need for a regulated turnover of NMJ components, which is thought to be mediated by their exocytic delivery and endocytic elimination [2]. A third recycling pool of previously surface-exposed receptors is apparently available for rapid recruitment upon NMJ activity-dependent demand [21], [22].

In search for the molecular machinery driving such vesicular transport, we looked for locomotion disorders involving vesicular transport proteins. Human Elejalde syndrome and Griscelli syndrome type 1 [23]–[25] as well as the rodent *dilute lethal* and *dilute opisthotonus* phenotypes [26], [27] are characterised by hypomelanosis, severe seizures, opisthotonus and premature death and are due to a lack of functional myosin Va. The molecular motor protein myosin Va is a processive [28], unconventional myosin with a broad tissue expression pattern [29] and is known to be involved in the transport of many vesicular carriers including skin pigment granules [30]–[32], neuronal [33], [34] and neuroendocrine vesicles [35]. In skin, myosin Va was shown to be important for capturing pigment granules in the peripheral F-actin-rich cortex in the dendritic tips of melanocytes [32]. In the absence of functional myosin Va, such as upon expression of a dominant-negative version of the motor (MCLT) or in melanocytes from *dilute lethal* mice, pigment granules were not captured in the cell cortex, but accumulated at the site of highest microtubule density, i.e. in the perinuclear region [30], [32].

In the present study we addressed the question of whether myosin Va is involved in maintenance and integrity of the postsynaptic apparatus of the NMJ. We show clear *in vivo* and *in vitro* evidence that myosin Va is in fact essential for long-term homeostasis of the mouse NMJ and that it may be particularly important for the recycling of AChR- and rapsyn-containing vesicles.

## Results

### DLS/LeJ mice exhibit severe postnatal degeneration of NMJs

**Figure 1:**
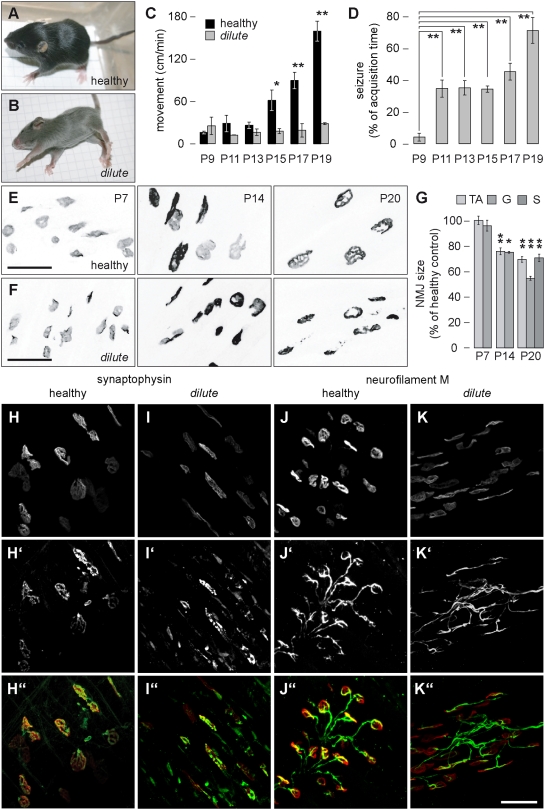
Aetiopathology and morphological deterioration of NMJs are temporally correlated in DLS/LeJ mice. A–D: Motility behaviour of healthy and *dilute* littermates was monitored from P9 to P19 using video analysis. A–B: Habitus of healthy and *dilute* littermates (indicated), at P14. Note light fur colour and convulsions in B. C: Quantification of unrestrained movement activity of healthy and *dilute* animals in a cage at the indicated ages. Columns, mean activity±s.e.m. (n = 6 and n = 3 healthy and *dilute* mice, respectively, from 3 different litters). D: Quantification of the fraction of observation time during which *dilute* animals showed clear signs of seizure at the indicated ages. Columns, mean±s.e.m. (P9, n = 5, P13, n = 4; P15, n = 5; P17, n = 3; P19, n = 3 mice from at least 3 different litters). E–K″: Tibialis anterior (TA), gastrocnemius (G), soleus (S) and peronaeus muscles were taken from healthy and *dilute* littermates on P7, P14 or P20, sliced longitudinally and stained with BGT-AF647 (E–G) or co-stained with BGT-AF647 and antibodies against synaptophysin or neurofilament M (H–K″). E–F: Maximum z-projections of confocal images of BGT-AF647 fluorescence signals. Scale bars, 50 µm. G: Quantification of NMJ areas. Graph, average reduction of NMJ sizes in TA, G and S muscles in *dilute* as compared to healthy (set to 100%) littermates. Columns, mean±s.e.m. (P7, n = 6; P14, n = 10 for TA, n = 5 for G; P20, n = 4; all n-values, number of animals). H–K″: Maximum z-projections of confocal images from P20 peronaeus muscles showing signals of BGT-AF647 (H–K), synaptophysin (H′, I′), neurofilament M (J′, K′) or the overlay of BGT-AF647 and immunostaining (red and green, respectively; H″–K″). Co-localising signals in H″–K″, yellow. Scale bar in K″, 50 µm.

First, we addressed the question whether the absence of functional myosin Va affects the integrity of NMJs. Therefore, we took advantage of the DLS/LeJ mouse line which carries the recessive *dilute lethal Myo5a^d-l^* allele (homozygous leading to light fur colour, severe convulsions and death within three weeks after birth). In our experiments, we compared homozygous *Myo5a* and heterozygous *Myo5a/Myo5a^d-l^* (both exhibiting black fur colour and referred to as ‘healthy’, Fig. 1A, at postnatal day 14, P14) with homozygous *Myo5a^d-l^* siblings (light fur colour, referred to as *‘dilute’*, Fig. 1B, at P14) first with respect to aetiopathology during the first three postnatal weeks. Barring fur colour, no differences could be observed between healthy and *dilute* animals until the ninth postnatal day (P9). Body weights (not shown) and motility were similar in all littermates (Fig. 1C, Fig. S1A and B, Movie S1) and convulsions were practically absent (Fig. 1D and Fig. S1C). However, from P10 onwards, *dilute* animals ceased growing (not shown) and did not increase their voluntary movement activities as it was observed throughout for healthy littermates (Fig. 1C). In fact, most of the translocation of the *dilute* animals (Fig. 1C and Fig. S1A and B) was from now on due to increasingly severe and prolonged seizures (Fig. S1D and Movies S2 and S3). While initially (between P10 and P16) mainly the extremities' musculature seemed to be affected by spasms, at later time points (P17 through P21) the animals were seen to contract as a whole, round up and fall on their back, with extended extremities exhibiting spastic tremor. This was all in sharp contrast to healthy littermates which continued growing, increased their activity range (Fig. 1C, Fig. S1A and B, Movies S1, S2, S3) and behaved normally.

To investigate whether such externally visible aetiopathology would show an internal correlate in the synapses regulating muscle contraction, we extracted different hind limb muscles (tibalis anterior [TA], gastrocnemius [G], soleus [S] and peronaeus) from healthy and *dilute* siblings on P7, P14 or P20. Some of these muscles were sliced longitudinally and then stained with a fluorescent marker for AChRs, alpha-bungarotoxin (BGT) coupled to AlexaFluor (AF) 647 (BGT-AF647). Subsequently, we performed morphometric analysis of NMJs upon maximum z-projections of 3D stacks obtained with confocal microscopy as shown in Fig. 1E and F (gastrocnemius samples are depicted). Interestingly, while at P7 NMJs in healthy and *dilute* animals were quite similar in shape (simple plaques; Fig. 1E and F, left panels) and size (Fig. 1G), at P14 dilute NMJs were about 20% smaller than in the control (Fig. 1G) and apparently stopped their morphological maturation, which in wildtype muscles led to fenestration and arborisation of the NMJ structure (compare Fig. 1E and F, middle panels). The deterioration of *dilute* NMJs was yet more aggravated at P20: NMJs were often fragmented (Fig. 1F, right panel, Table 1) and their areas were further diminished (Fig. 1G). In summary, the aetiopathology of seizures appeared to correlate with the time course of morphological changes at the NMJ, suggesting that myosin Va might play a role in the maintenance and/or development of the NMJ.

**Table 1:** NMJ fragmentation in DLS/LeJ mice.

	P7 *dilute*	P7 healthy	P14 *dilute*	P14 healthy	P20 *dilute*	P20 healthy
Mean number of NMJ fragments per NMJ	1,06±0,02	1,03±0,02	1,15±0,05	1,02±0,01	2,52±0,13	1,11±0,04
Minimal number of fragments	1	1	1	1	1	1
Maximal number of fragments	3	2	5	2	7	3

Gastrocnemius muscles of healthy and *dilute* mice were explanted at P7, P14 or P20. Muscles were fixed, sliced and stained with BGT-AF647. Upon confocal imaging and image processing, the numbers of NMJ fragments identified as discontinuous gutters of each NMJ were counted. Data, mean±s.e.m. (n>100 analysed NMJs per condition from at least two different animals).

To rule out, that NMJ sizes simply followed a decrease in fibre diameter, we measured the latter characteristic for some muscles at P14 and P20. At P14, when NMJs of *dilute* muscles were already more than 20% smaller than the healthy ones, fibre diameters of TA muscles of healthy and *dilute* mice were identical with 23.0 µm±0.5 µm (mean±s.e.m., n = 45 fields, 521 fibres measured) and 23.0 µm±0.3 µm (mean±s.e.m., n = 46 fields, 502 fibres measured), respectively. Conversely, at P20 *dilute* TA muscles exhibited a significantly smaller mean fibre diameter (21.4 µm±2.4 µm, mean±s.e.m., n = 6 fields, 138 fibres measured) than their healthy counterparts (26.1 µm±2.4 µm, mean±s.e.m., n = 12 fields, 195 fibres measured). These results clearly show that the shrinkage of NMJ areas in the absence of functional myosin Va occurs before and independently of the decrease in fibre diameter. Next, we tested whether shrinkage and fragmentation observed at the postsynaptic side were due to a lack of innervation. Therefore, we studied the innervation status by immunolabelling the presynaptic proteins, synaptophysin and neurofilament M, in muscles from *dilute* and healthy littermates at P20. The synaptophysin signals nicely matched the AChR signals in healthy mice (Fig. 1H–H″) as well as *dilute* mice (Fig. 1I–I″). Importantly, each postsynaptic AChR cluster clearly was at close range to presynaptic synaptophysin, and each endplate was contacted by a single nerve ending as evidenced by neurofilament M staining (Fig. 1J–K″). It should be noted, that the morphological alterations of the postsynapses in *dilute* mice (Fig. 1I and K) were mirrored onto the presynapses (Fig. 1I′ and K′), leading to a clearly different morphology of the nerve branches when compared to healthy synapses. In particular, the neurofilament staining in *dilute* muscles did in most cases not embrace the synaptic contacts as nicely as in healthy mice, but rather continued linearly into the elongated postsynaptic area (Fig. 1K–K″). To verify whether the presynaptic markers were present in comparable amounts in healthy and *dilute* muscles, we undertook a statistical analysis of the BGT-stained areas covered by either synaptophysin or neurofilament M staining (Table 2). This revealed that both marker proteins covered about 60% of the postsynaptic area in wildtype as well as in *dilute* muscles. To make sure that these synapses were still capable of nerve transmission, we elicited hind limb muscle contraction through sciatic nerve electrodes in live anaesthetised mice at P14. Under these conditions the stimulated muscles of all healthy and *dilute* animals clearly contracted (not shown). Altogether, these data indicate that *dilute* muscles are innervated and that synapses are still able to induce muscle contraction, suggesting that the altered postsynaptic morphology in these mice was not due to missing innervation.

**Table 2:** Coverage of BGT-positive area by presynaptic markers in healthy and dilute muscles.

marker	healthy	*dilute*
synaptophysin	66.3±5.1 (4)	63.4±1.6 (3)
neurofilament M	59.0±3.5 (8)	60.7±4.4 (8)

Peronaeus muscles of healthy and *dilute* mice were explanted at P19. Muscles were fixed, sliced and co-stained with postsynaptic BGT-AF647 and antibodies against presynaptic synaptophysin or neurofilament M. Upon confocal microscopy and preparation of maximum z-projections, co-localisation between post- and presynaptic markers was determined. Data show the average fraction of the BGT-AF647-positive area in individual NMJs, which was covered by synaptophysin or neurofilament M immunofluorescence signals in percent ±s.e.m. (n-values, number of analysed microscopic fields). The number of analysed NMJs was >40 from at least two different muscles for each condition.

### Rapsyn-GFP containing vesicular structures are enriched in the perinuclear region of FLAG-MCLT transfected myotubes

**Figure 2:**
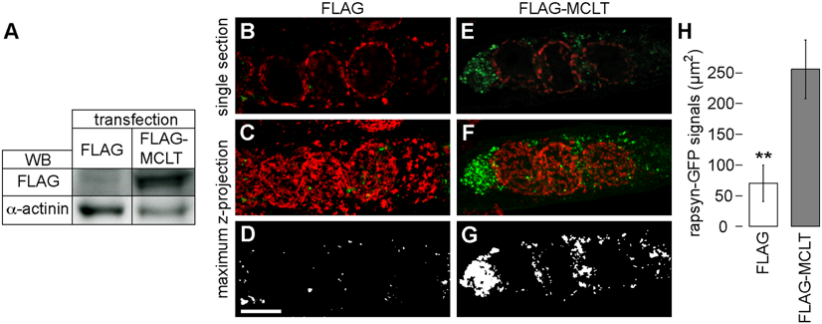
Rapsyn-GFP accumulates in the perinuclear regions of FLAG-MCLT expressing C2C12 myotubes. C2C12 myoblast cells were co-transfected with rapsyn-GFP and FLAG (FLAG) or FLAG-MCLT (FLAG-MCLT). Upon seven days of differentiation, cells were lysed (A) or fixed and immunostained for GM130 (B–H). A: Bands, western blot signals upon incubation with FLAG or alpha-actinin antibodies of lysates of cells transfected with FLAG or FLAG-MCLT (indicated). B, E: Optical sections of rapsyn-GFP (green) and GM130 (red). C, F: Maximum z-projections of the cells depicted in B and E. D, G: Binarised rapsyn-GFP signals from projections shown in C and F for quantification of rapsyn-GFP positive areas. Scale bar, 10 µm. H: Quantification of total rapsyn-GFP positive area per myotube on binarised maximum z-projections. Data, mean±s.e.m. (n = 5 experiments).

To further support the hypothesis that myosin Va is important for the transport of NMJ components in the absence of neuronal and glial tissues, we next used the C2C12 cell model system. C2C12 myoblasts were co-transfected with the NMJ marker protein, rapsyn-GFP, and FLAG or FLAG-MCLT. FLAG-MCLT is a FLAG-tagged form of MCLT (myosin Va C-terminal long tail) and was previously shown to act as dominant negative fragment of myosin Va in different cellular models [32], [36]. Its expression in C2C12 cells was verified by Western blot (Fig. 2A). Transfected cells were differentiated into myotubes for seven days, fixed, immunostained for the cis-Golgi marker, GM130, and then visualised using confocal microscopy. As shown in Fig. 2B–D, rapsyn-GFP was localised in punctate structures distributed evenly all over the myotubes in the absence of FLAG-MCLT. Conversely, in FLAG-MCLT transfected myotubes, rapsyn-GFP positive punctate structures were highly enriched (Fig. 2E–H) and mostly concentrated in perinuclear clusters (Fig. 2E–G, the relative orientation between GM130 and nuclei is depicted in Fig. S2). It is unlikely that the effects on amount and localisation of rapsyn-GFP positive structures observed in FLAG-MCLT co-transfected myotubes were due to general morphological alterations, since both FLAG and FLAG-MCLT co-transfected myotubes showed the typical pearl chain arrays of ring-like Golgi apparatuses (compare Fig. 2B–C and E–F). These data suggest that myosin Va has an impact on the distribution of carriers containing NMJ components.

### Myosin Va co-precipitates with surface-exposed or internalised AChRs

**Figure 3:**
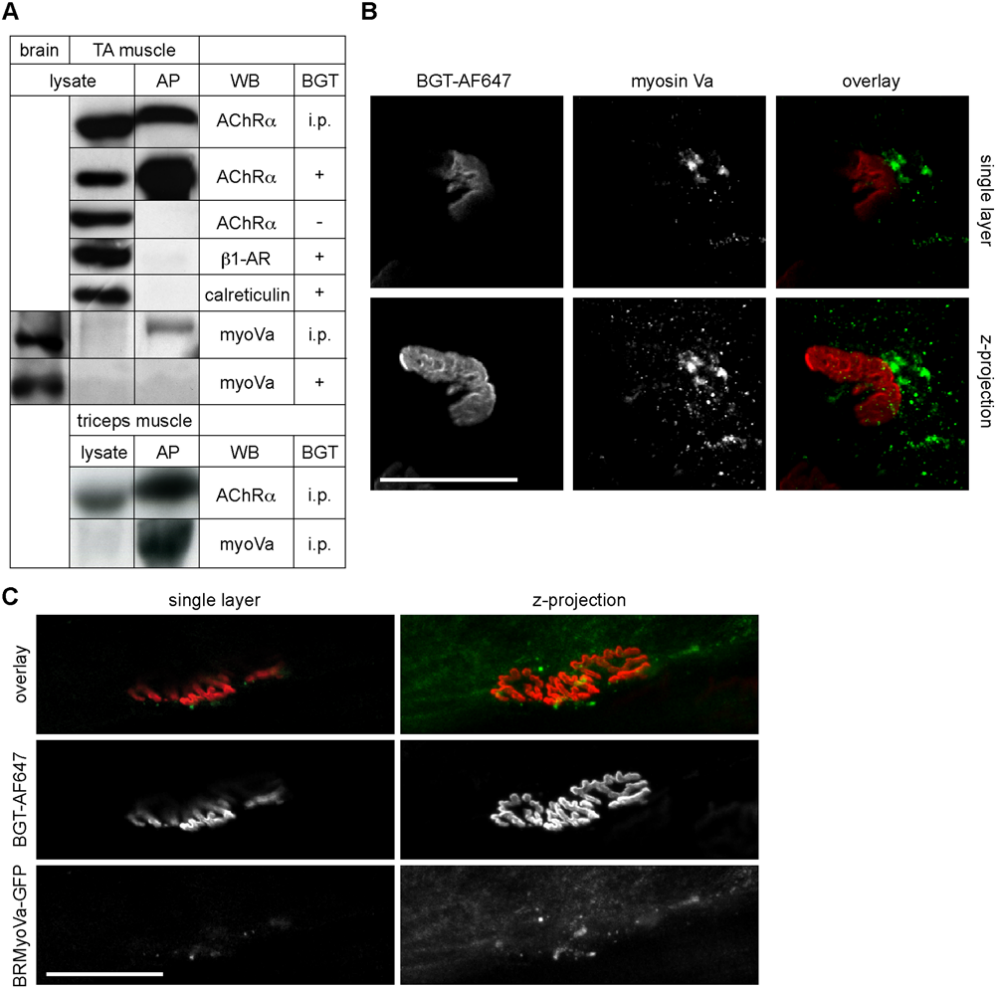
Myosin Va is enriched in punctate structures in the postsynaptic region of skeletal muscle. A: Wildtype mice were intraperitoneally injected with BGT-biotin (BGT, i.p.) or left untreated. Then, lysates of brain as well as TA and triceps muscles (indicated) were prepared. Samples from untreated mice were incubated in the presence or absence of BGT-biotin (BGT, + or −, respectively). Then, all samples were incubated with NeutrAvidin beads and AChRs affinity precipitated. Bands, representative Western blot signals from lysates and affinity precipitates (AP) using antibodies against proteins as indicated (WB). B: Triceps muscles of wildtype mice (P20) were co-stained with BGT-AF647 and LF-18 myosin Va antibody and then imaged with confocal microscopy. Single confocal layer and corresponding maximum z-projection of 43 background-subtracted optical slices taken at 0.7 µm interslice distance encompassing the entire extension of the shown NMJ in depth. Overlay, BGT-AF647 (red), myosin Va antibody signal (green). Scale bar, 50 µm. C: TA muscles of adult wildtype mice were transfected with full length brain myosin Va-GFP (BRMyoVa-GFP). Ten days later, transfected muscles were locally injected with BGT-AF647 and then imaged with *in vivo* confocal microscopy. Single layer and corresponding maximum z-projection of 29 optical slices taken at 0.7 µm interslice distance encompassing the entire extension of the shown NMJ in depth. Overlay, BGT-AF647 (red), myosin Va-GFP (green). Scale bar, 50 µm.

Next we addressed the presence and localisation of endogenous myosin Va in mouse muscle. Therefore, we first performed affinity co-precipitation assays, in which we aimed to pull down different AChR populations. In one approach, we used intraperitoneal (i.p.) injection of BGT-biotin into live mice. Since BGT is cell-impermeant, we expected to label by this means only surface-exposed and internalised (endocytosed and recycling) AChRs. In another experimental set, we added BGT-biotin to whole muscle lysates of untreated mice to label the total population of AChRs [37]. Subsequently, using NeutrAvidin resin, BGT-biotin-labeled AChRs in all these samples were harvested by affinity precipitation (AP) and co-precipitating proteins were detected by Western blot analysis. AChR alpha-subunit precipitated only in the presence but not the absence of BGT-biotin (Fig. 3A, compare rows 1–2 and 3), showing that AChRs did not bind unspecifically to the resin. Furthermore, two proteins known to not be associated with AChRs, namely beta1-adrenergic receptor and calreticulin, could not be found in the precipitates even in the presence of BGT-biotin (Fig. 3A, rows 4–5), indicating again the specificity of the pull-down assay. Using myosin Va antibody, we found strong signals in brain lysates and precipitates from muscle samples where BGT-biotin was i.p. injected, but no or barely visible signals were present in muscle lysates (Fig. 3A, row 6). A similar enrichment of myosin Va in AChR precipitates was detected also in other muscles, such as in triceps muscle (Fig. 3A, row 9). Interestingly, the accumulation of myosin Va was only observed in muscle samples where BGT-biotin was i.p. injected (Fig. 3A, rows 6 and 9), but not when BGT-biotin was added to the whole muscle lysate (Fig. 3A, row 7). This suggests, that myosin Va is mostly associated with either surface-exposed or internalised AChRs, but hardly with newly synthesised AChRs transported from the endoplasmic reticulum to the NMJ.

### Myosin Va is enriched in the vicinity of the NMJ

To study the localisation of endogenous myosin Va in skeletal muscle, we performed immunofluorescence stainings. Muscles from young wildtype mice were briefly fixed, labeled with antibodies for myosin Va (LF-18 or ham5) and BGT-AF647 for AChRs and then analysed with confocal microscopy. As depicted in Fig. 3B for a triceps muscle stained with LF-18 antibody, myosin Va signals were detected in most fibres as small punctate structures and as larger aggregates in close proximity to the NMJ. Movie S4 shows a three-dimensional view of peronaeus muscle stained with ham5 antibody and BGT-AF647. Controls processed without primary antibody did not show any sign of punctate or aggregated signals using the same microscope settings (not shown). To further confirm the association of myosin Va with structures bearing NMJ components, we transfected full length myosin Va fused to GFP into TA muscles of adult wildtype mice employing *in vivo* muscle transfection and *in situ* confocal microscopy essentially as described previously [38]–[40]. Ten days after transfection, BGT-AF647 was locally injected into muscles and then myosin Va-GFP and BGT-AF647 were simultaneously monitored by *in vivo* confocal microscopy. Fig. 3C shows a single layer and a corresponding maximum z-projection of a 3D stack encompassing the entire extension of a representative NMJ. The myosin Va-GFP fluorescence signals were detected in small, punctate structures closely localised to the NMJ, whereas outside the perijunctional area the GFP-staining appeared diffuse.

### Myosin Va tail co-localises with NMJ marker proteins in punctate structures

**Figure 4:**
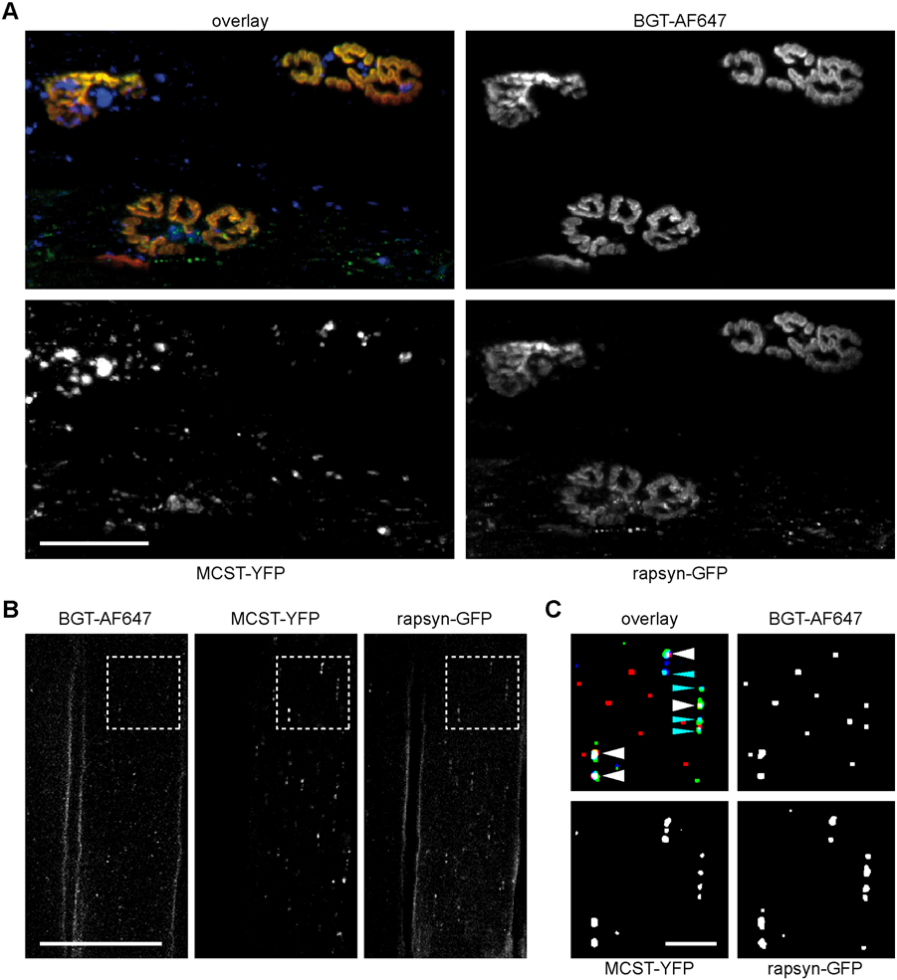
Myosin Va tail is enriched in punctate structures in the postsynaptic region of NMJs and co-localises with AChRs and rapsyn-GFP. TA muscles of adult wildtype mice were co-transfected with rapsyn-GFP and MCST-YFP. Ten days later, transfected muscles were locally injected with BGT-AF647 and then imaged with *in vivo* confocal microscopy. Overlay images, BGT-AF647 (red), rapsyn-GFP (green), MCST-YFP (blue). A: Maximum z-projection of 42 optical slices taken at 2 µm interslice distance encompassing the entire extension of shown NMJs in depth. Scale bar, 50 µm. B: Single layer through the center of a fibre. Scale bar, 50 µm. C: Binarised blow-up of the boxed region in (B). Overlay, punctate structures triple positive for BGT-AF647, rapsyn-GFP and MCST-YFP, white arrowheads; double positive for rapsyn-GFP and MCST-YFP, blue arrowheads. Scale bar, 10 µm.

Next, we tested the localisation of a short C-terminal fragment of myosin Va fused to YFP upon co-expression with rapsyn-GFP and injection of BGT-AF647 in adult wildtype muscle using *in vivo* confocal microscopy as before. Fusion constructs of MCST and fluorescent proteins have been previously shown to bind to cargo vesicles which were also transported by endogenous full-length myosin Va [32]. Fig. 4A shows the maximum z-projection of a 3D stack encompassing the entire extension of the depicted NMJs. In the presence of MCST-YFP, NMJs exhibited a rather normal, unfragmented appearance and rapsyn-GFP perfectly co-localised with BGT-AF647 signals (Fig. 4A). Notably, the localisation of MCST-YFP was again quite similar to that of endogenous myosin Va, i.e. it was found to be enriched in many punctate structures and aggregates of variable size in the immediate vicinity of NMJs (Fig. 4A). Having found that the MCST-YFP distribution mimicked that of endogenous myosin Va, we next asked whether individual MCST-YFP-positive punctate structures contained also AChRs and/or rapsyn-GFP. Upon BGT-AF647 injection we took *in vivo* confocal images using enhanced detector gains and observed muscle fibres expressing MCST-YFP and rapsyn-GFP at apparently similar levels. Special care was taken to avoid any signal crosstalk. As depicted in Fig. 4B–C, most MCST-YFP positive puncta were either double-positive for rapsyn-GFP (Fig. 4C, blue arrowheads) or triple-positive for rapsyn-GFP and BGT-AF647 (Fig. 4C, white arrowheads). A quantitative analysis of image stacks from three different animals revealed that on average 76.3%±2.5% (mean±s.e.m.; 956 punctate structures from n = 5 image stacks were analysed) of MCST-YFP positive puncta also contained rapsyn-GFP and BGT-AF647. In the same data sets 9.2%±3.6% or 8.5%±1.0% of MCST-YFP positive puncta displayed only either rapsyn-GFP or BGT-AF647 fluorescence, respectively (all values, mean±s.e.m.). Together with the affinity precipitation (Fig. 3A) and myosin Va immunofluorescence results (Fig. 3B and Movie S4), the full length myosin Va-GFP (Fig. 3C) and MCST-YFP *in vivo* localisation data (Fig. 4) strongly indicate that myosin Va is enriched in the region of the NMJ and suggest that the motor protein interacts directly or indirectly with the AChR.

### The sizes of NMJs are reduced upon inhibition of myosin Va function

**Figure 5:**
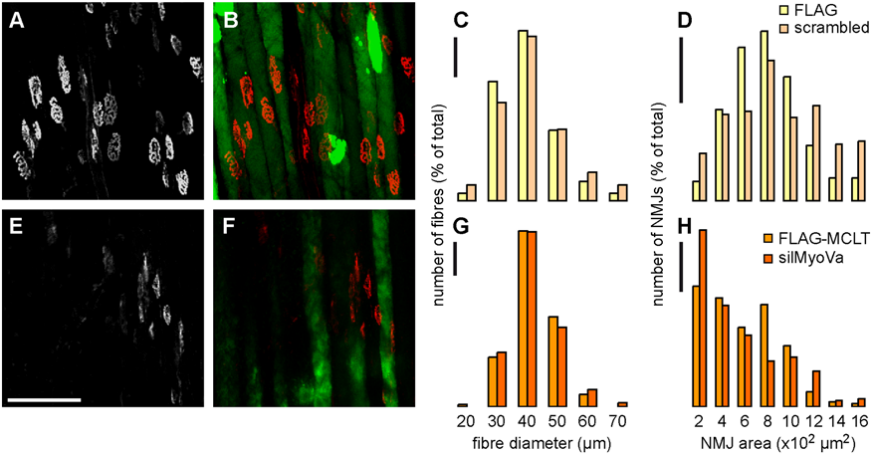
NMJs shrink upon transfection with FLAG-MCLT or silMyoVa in vivo. TA muscles of adult wildtype mice were co-transfected with cytoplasmic YFP and FLAG (A–D), FLAG-MCLT (E–H), scrambled silMyoVa (C–D) or silMyoVa (G–H). Ten days later, transfected muscles were locally injected with BGT-AF647 and imaged with *in vivo* confocal microscopy. A–B and E–F: Representative maximum z-projections of 74 and 64 optical slices in A–B and E–F, respectively, taken at 3 µm interslice distance. A and E: Signals of BGT-AF647. Scale bar, 200 µm. B and F: Overlay of BGT-AF647 (red) and cytoplasmic YFP (green). C–D and G–H: Histograms, fibre diameter distribution (C, G) or NMJ area distribution (D, H) for the same set of fibres. Scale bars, 10% of total amount of measured fibres or NMJs. For each condition, at least 202 fibres or NMJs from at least 4 different animals were analysed.

Next we tested the effect of functional ablation of myosin Va on the size of the NMJ. To that end, TA muscles of adult wildtype mice were co-transfected with a cytoplasmic YFP probe (to measure the fibre diameter) and either FLAG-MCLT or a construct encoding siRNA against myosin Va (silMyoVa). The latter was shown in neuroendocrine PC12 cells to significantly reduce myosin Va levels and to lead to blockage of myosin Va functions as through the action of FLAG-MCLT (Kögel et al., unpublished). As controls, TA muscles were co-transfected with a cytoplasmic YFP probe and FLAG, or a scrambled siRNA construct. Ten days later, muscles were injected with BGT-AF647 and then visualised using *in vivo* confocal microscopy. Maximum z-projections of background-subtracted optical slices showing BGT-AF647 signals (Fig. 5A and E) were prepared and the sizes of NMJs determined. The fibre diameter corresponding to any measured NMJ was taken at the widest extension of the fibre in the cytoplasmic YFP images (Fig. 5B and F). The distributions of fibre diameters and NMJ sizes are shown in the histograms of Fig. 5C and G and Fig. 5D and H, respectively. Comparing the controls with the animals transfected with either FLAG-MCLT or silMyoVa, there was no significant difference with regard to fibre diameter (Fig. 5C and G). Under all conditions, the diameters ranged in a binomial distribution from 20 to 70 µm with a clear peak at 40 µm. Conversely, NMJ areas exhibited very different distributions in controls and myosin Va impeding conditions (Fig. 5D and H). While in the controls NMJ sizes showed a roughly bell-shaped distribution with a peak at 800 µm^2^, NMJ areas were much smaller in the presence of either FLAG-MCLT or silMyoVa, now showing a peak at 200 µm^2^. Average NMJ areas and fibre diameters are given in Table 3. These data strongly suggest that NMJs shrink upon inhibition of myosin Va in live adult wildtype mice, and that this diminution is not due to a decrease in fibre diameter. Furthermore, immunostaining of presynapses and muscle force recordings upon muscle or nerve stimulation provide evidence that the transfected muscles were innervated properly (Fig. S3).

**Table 3:** Average NMJ areas and fibre diameters upon functional impairment of myosin Va.

	FLAG	FLAG-MCLT	Scrambled silMyoVa	silMyoVa
NMJ area (µm^2^)	706.8±22.7	475.7±18.6**	753.4±28.2	426.5±23.3**
Fibre diameter (µm)	35.1±0.6	36.8±0.4	36.2±0.7	37.4±0.5
Number of fields (animals)	8 (6)	8 (4)	7 (4)	9 (4)

TA muscles of adult wildtype mice were co-transfected with cytoplasmic YFP and FLAG, FLAG-MCLT, scrambled silMyoVa or silMyoVa, as indicated. Ten days later, transfected muscles were locally injected with BGT-AF647 and imaged with *in vivo* confocal microscopy. NMJ areas and diameters of corresponding fibres were determined. Data, mean±s.e.m.

### NMJs become fragmented and show a high turnover of AChRs in muscles transfected with silMyoVa

**Figure 6:**
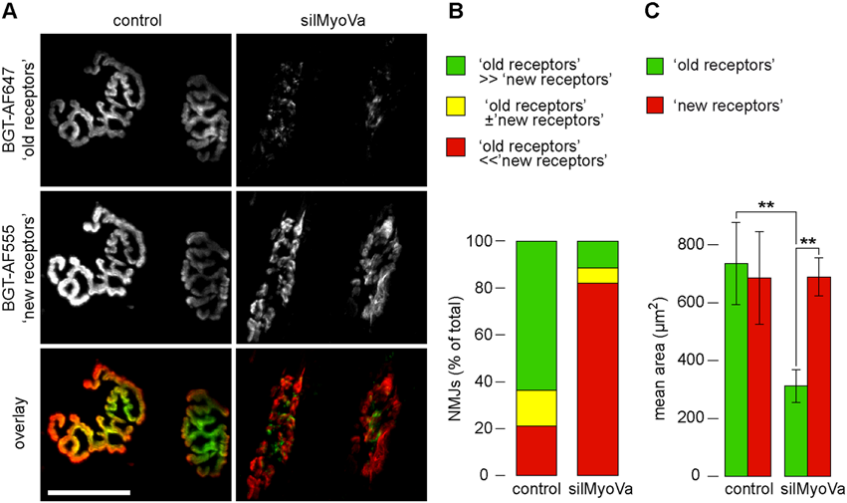
Persistence of AChRs in NMJs is reduced in muscles transfected with silMyoVa in vivo. TA muscles of adult wildtype mice were either transfected with rapsyn-GFP (control) or co-transfected with rapsyn-GFP and silMyoVa (silMyoVa). Simultaneously, muscles were locally injected with BGT-AF647. Ten days later, transfected muscles were locally injected with BGT-AF555 and then imaged using *in vivo* confocal microscopy. A: Maximum z-projections of 13 (control) and 7 (silMyoVa) optical slices taken at 2 µm interslice distance encompassing the entire extensions of shown NMJs in depth. Overlay image, BGT-AF647 (green), BGT-AF555 (red), co-localisation (yellow). Scale bar, 50 µm. B: Quantification of NMJs exhibiting a ratio of BGT-AF647 to BGT-AF555 volume signals smaller than 0.8 (‘old receptors’≪‘new receptors’), 0.8–1.2 (‘old receptors’±‘new receptors’) or higher than 1.2 (‘old receptors’≫‘new receptors’). Data are from 34 (control) and 61 (silMyoVa) NMJs (4 animals per condition). C: Determination of BGT-AF647 (‘old receptors’) and BGT-AF555 (‘new receptors’) positive areas in individual NMJs. Data, mean±s.e.m. (control, n = 15 NMJs; silMyoVa, n = 24 NMJs, from 2 different animals each).

Next we asked in a kind of pulse-chase experiment, which pool of AChRs (i.e. either newly synthesised or internalised) is affected by inhibition of myosin Va. Therefore, TA muscles of adult wildtype mice were either transfected with rapsyn-GFP (control) or co-transfected with rapsyn-GFP and silMyoVa (silMyoVa). During transfection, BGT-AF647 was injected locally. Ten days later, transfected muscles were injected with BGT-AF555. Then, BGT-AF647 and BGT-AF555 fluorescence signals (Fig. 6A, green and red in overlay images, respectively) were monitored by *in vivo* confocal microscopy. Given the extreme stability of the AChR-BGT complex [41] and the small quantities of BGT (25 pmol) injected at each occasion, we reasoned that the largest proportion of BGT should have been available in the circulatory system only for a limited amount of time and therefore AChRs marked by BGT should have been surface-exposed at the time point of injection, i.e. ten days or about one hour before inspection for BGT-AF647 or BGT-AF555, respectively. Indeed, spectrophotometric measurements suggest that most BGT is available for binding only during the first few hours upon injection (not shown). In control muscles, the two BGT fluorescence signals showed a very nice overlap and clearly depicted the typical arborised structure of adult NMJs (Fig. 6A, left panels). There was, however, a gradient of intensities: while BGT-AF647 (referred to as ‘old receptors’) was preferably localised in the NMJ centre (Fig. 6A, green in overlay images), BGT-AF555 (referred to as ‘new receptors’) showed a higher concentration at the rims of NMJs (Fig. 6A, red in overlay images). Upon co-transfection with silMyoVa, the overall architecture of NMJs was significantly altered and appeared fragmented (Fig. 6A, right panels). Furthermore, although intensity and extension of ‘new receptors’ were still comparable to control muscles (Fig. 6A, compare central panels), ‘old receptors’ were almost completely gone in silMyoVa transfected muscles (Fig. 6A, compare upper panels). Finally, as in the control, in silMyoVa transfected muscles ‘old receptors’ were found in the centre and ‘new receptors’ at the rim of NMJs. However, in most cases there was now an almost net separation of the domains of ‘old receptors’ and ‘new receptors’ (Fig. 6A, bottom right panel).

Quantitative analyses measuring volume (Fig. 6B) and areas (Fig. 6C) of fluorescence signals of ‘new receptors’ and ‘old receptors’ supported these notions. In Fig. 6B those NMJs were grouped which showed a ratio of ‘old receptor’ to ‘new receptor’ volume signals lower than 0.8 (Fig. 6B, ‘old receptors’≪‘new receptors’), between 0.8 and 1.2 (Fig. 6B, ‘old receptors’±‘new receptors’) or higher than 1.2 (Fig. 6B, ‘old receptors’≫‘new receptors’). It is evident that the signals of ‘old receptors’ exceeded those of ‘new receptors’ in most NMJs in control conditions (Fig. 6B, ‘old receptors’≫‘new receptors’). Conversely, upon transfection with silMyoVa, most NMJs showed a smaller volume of ‘old receptor’ than of ‘new receptor’ signals. When concentrating on the areas occupied by ‘old receptors’ and ‘new receptors’, transfection of silMyoVa apparently led in first instance to a strong reduction in the ‘old receptor’ signals, while ‘new receptor’ signals remained unaltered (Fig. 6C). In summary, these data indicate that interference with myosin Va function primarily decreases the persistence of receptors at the NMJ whereas the delivery of ‘new receptors’ is less affected.

### Rapsyn-GFP is mislocalised and enriched in BGT-positive punctate structures close to the NMJ in live muscle transfected with FLAG-MCLT or silMyoVa

**Figure 7:**
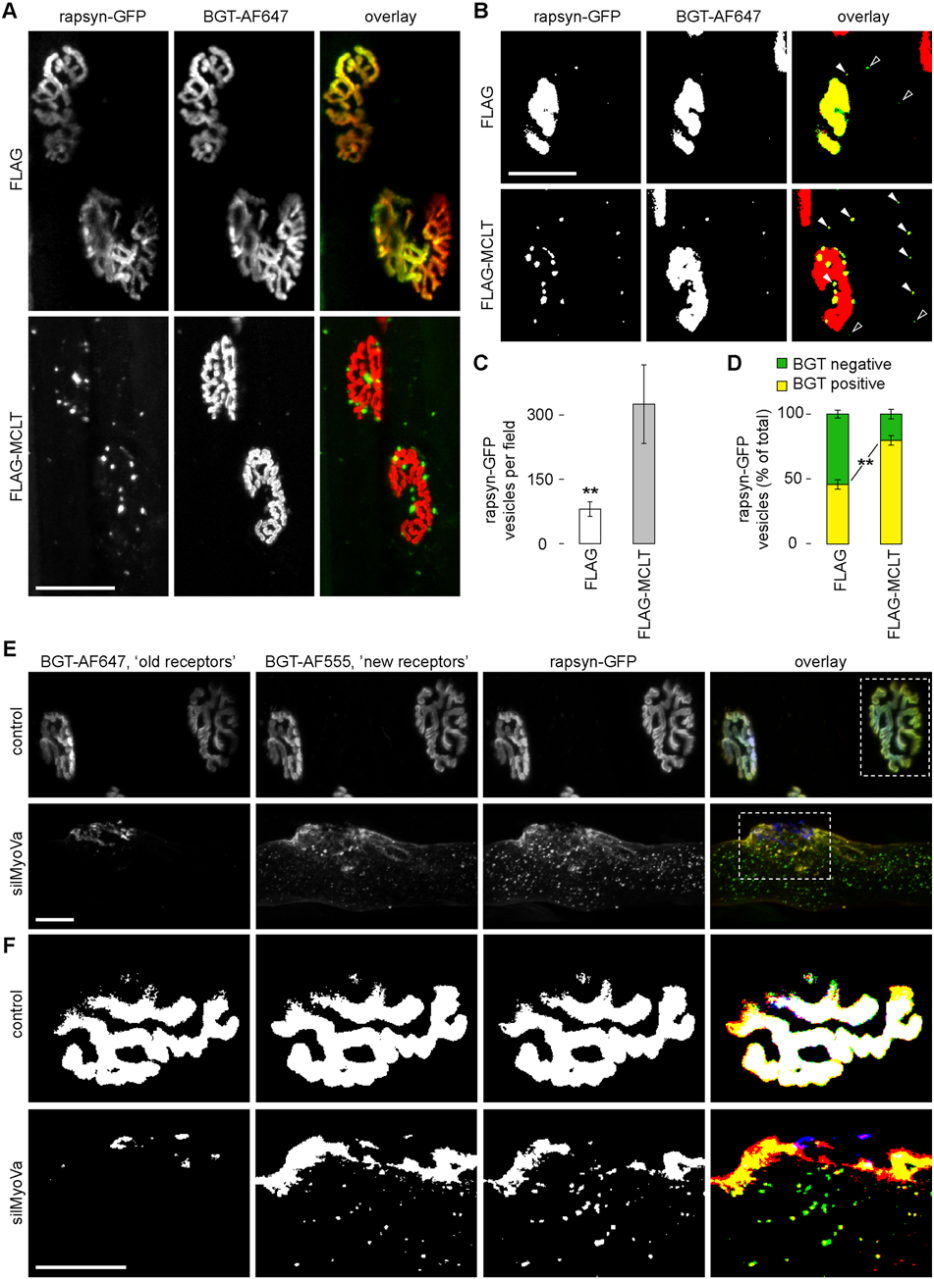
Amount of rapsyn-GFP positive punctate structures and their co-localisation with BGT-AF647 are increased in FLAG-MCLT or silMyoVa transfected muscles in vivo. TA muscles of adult wildtype mice were either transfected with rapsyn-GFP (E–F) or co-transfected with rapsyn-GFP and FLAG (A–D), FLAG-MCLT (A–D) or silMyoVa (E–F). Muscles in E–F were also injected with BGT-AF647 during transfection. Ten days later, muscles were locally injected with BGT-AF647 (A–D) or BGT-AF555 (E–F) and imaged with *in vivo* confocal microscopy. A–B: Overlay images, rapsyn-GFP (green), BGT-AF647 (red), co-localising signals (yellow). Scale bar, 50 µm. A: Maximum z-projections of 80 (FLAG) and 37 (FLAG-MCLT) optical slices taken at 1.5 and 1 µm interslice distance, respectively, encompassing the entire extensions of shown NMJs in depth. B: Single optical sections binarised with equal thresholds upon background subtraction for better visibility. Rapsyn-GFP vesicular structures positive and negative for BGT-AF647 in the overlay image, white-filled and empty arrowheads, respectively. C: Quantification of rapsyn-GFP vesicular structures per microscopic field (≥12 per condition). Data, mean±s.e.m. (n = 3 mice). D: Quantification of BGT-AF647 positive and negative rapsyn-GFP vesicular structures. Data, mean±s.e.m. (n = 3 mice, ≥12 microscopic fields per condition, 1112 (FLAG) and 3982 (FLAG-MCLT) analysed vesicular structures). E–F: Overlay images, rapsyn-GFP (green), BGT-AF555 (red), BGT-AF647 (blue). Scale bars, 25 µm. E: Maximum z-projections of 33 (control) and 53 (silMyoVa) optical slices taken at 0.8 and 1 µm interslice distance, respectively, encompassing the entire extensions of shown NMJs in depth. F: Single optical slices binarised with equal thresholds upon background subtraction for better visibility through the centre of shown NMJs. Overlay of rapsyn-GFP and BGT-AF555 (yellow), of BGT-AF555 and BGT-AF647 (magenta), and of rapsyn-GFP, BGT-AF555 and BGT-AF647 (white).

The experiments so far have delivered evidence that myosin Va is localised close to the NMJ, that the interference with the motor protein's function induces shrinkage of the NMJ and that this shrinkage is likely due to a reduced persistence of AChRs at the NMJ. To better understand the fate of these receptor molecules, we looked for the enrichment of putative endocytic structures upon blockage of myosin Va. Therefore, we expressed rapsyn-GFP, which should be present in both exocytic as well as endocytic vesicles, together with FLAG or FLAG-MCLT. Ten days after transfection muscles were locally injected with BGT-AF647. Then, they were exposed for *in vivo* microscopy and rapsyn-GFP and BGT-AF647 fluorescence signals were monitored simultaneously. As depicted in Fig. 7A, rapsyn-GFP overlapped with the BGT-stained AChRs in FLAG-transfected muscles (upper panels), but was largely excluded from the NMJs of most fibres upon expression of FLAG-MCLT (lower panels). In the latter, rapsyn-GFP was highly enriched in punctate structures in close proximity to the NMJ and the amount of rapsyn-GFP positive structures discontinuous with the NMJ was highly increased (Fig. 7B and C). To get a hint on the nature of these puncta, we visualised the BGT-AF647 fluorescence signals in more detail. Given that BGT-AF647 as a cell-impermeant marker can bind AChRs only upon their exposure to the cell surface, intracellular BGT-positive structures should represent endocytic or internalised objects [42]. Remarkably, already one to two hours after BGT injection such intracellular BGT-positive structures were highly enriched in FLAG-MCLT co-transfected muscles (Fig. 7B, note filled and open arrowheads), similar to the situation for rapsyn-GFP. Furthermore, the extent of co-localisation of rapsyn-GFP with BGT-AF647 positive structures increased from 45.9%±3.0% to 79.9%±3.7% (both mean±s.e.m., n = 3 animals) in FLAG and FLAG-MCLT co-transfected muscles, respectively (Fig. 7D). These *in vivo* data strongly suggest that myosin Va is important for the localisation of rapsyn-GFP and that it is likely implicated in processes concerning internalisation of NMJ components.

Finally, we also addressed the question, from which pool of AChRs rapsyn-GFP was excluded upon interference with myosin Va function. Muscles were either transfected with rapsyn-GFP (control) or co-transfected with rapsyn-GFP and silMyoVa (silMyoVa) and were simultaneously injected with BGT-AF647. Ten days later, muscles were injected with BGT-AF555. Subsequently, BGT-AF647 (‘old receptors’), BGT-AF555 (‘new receptors’) and rapsyn-GFP signals were monitored with *in vivo* confocal microscopy. Fig. 7E depicts 3D projections of 33 and 53 optical slices of control and silMyoVa transfected muscle fibres, respectively, taken at 1 µm interslice distance (for rotating 3D views, see Movies S5 and S6). In control muscles rapsyn-GFP, ‘old receptors’ and ‘new receptors’ appeared co-localised in the NMJ. The co-localisation was, however, abolished in silMyoVa transfected fibres and, in particular, the ‘old receptors’ and ‘new receptors’ occupied distinct regions in the postsynapse (Fig. 7E and F). Furthermore, while in the silMyoVa transfected condition BGT-AF647 puncta were virtually absent, much more BGT-AF555 positive puncta were visible than in the control. Interestingly, most of these BGT-AF555 signals co-localised with rapsyn-GFP (Fig. 7E–F, lower panels). These results confirmed the findings of the previous experiments. Further, since BGT-AF555 was injected into the muscle shown in Fig. 7E–F only 90 minutes prior to image acquisition these data suggested that newly arrived AChRs and rapsyn-GFP were internalised at a high rate. Assuming similar internalisation rates of control and myosin Va inhibited muscles, this could indicate an inefficient recruitment of endocytosed AChRs in the presence of FLAG-MCLT or silMyoVa back to the postsynaptic membrane.

## Discussion

Depending on strain and physiological status of a muscle, neuromuscular junctions (NMJs) may undergo significant changes in size and shape [7], [43], [44]. In order to maintain the overall structure of the NMJ intact and yet allow for the necessary plasticity to occur, an intriguing homeostasis of vesicular exocytic, endocytic and recycling transport processes is thought to act [21]. We set out to identify the molecular machineries driving these dynamic processes. Here, we provide *in vivo* and *in vitro* evidence for a crucial role of the molecular motor protein, myosin Va, in the postsynaptic plasticity of the mammalian nerve-muscle synapse.

First, we show that NMJs in *dilute lethal* mice (referred to as ‘*dilute* mice’ in the following) lacking functional myosin Va exhibit a postnatal, time-dependent morphological deterioration that correlates with the dramatic aetiopathology leading to lethal seizures at around P21 (Fig. 1, Fig S1, Movies S1, S2, S3). Initial mild locomotor symptoms at around P13 coincided with the reduction of NMJ areas in *dilute* homozygotes. Seven days later, when homozygotes showed most severe convulsions, NMJs were in addition often fragmented. These postsynaptic modifications were apparently not due to a lack of innervation, since the *dilute* just as the control muscles exhibited clear presynaptic terminals and responded to nerve stimulation with contraction. Given that in wildtype animals the NMJ size was found to positively correlate with muscle fibre diameter [44], we also investigated whether fibre and NMJ sizes correlated with each other. These measurements revealed that NMJ size reduction preceded diminution of fibre diameters, indicating that, at least in *dilute* mice, fibre size reduction is a secondary effect. This might either be caused by an altered NMJ activity or by other, unrelated processes. At present, it is unclear, whether our observation of fragmented and small NMJs in *dilute* mice could partially explain their severe locomotor defects, in particular because previously also a correlation between aetiopathology and functional defects in the cerebellum of myosin Va deficient mice and rats was described [27], [45], [46], [but see for conflicting results 47], [48]. Altogether, our data on *dilute* mice suggest a role of myosin Va in postnatal NMJ maintenance and/or plasticity.

Our experiments using differentiating C2C12 cells (Fig. 2 and S2) co-transfected with the NMJ marker protein rapsyn-GFP [5] and a dominant-negative C-terminal fragment of myosin Va (FLAG-MCLT) showed an accumulation of the NMJ marker in perinuclear clusters. At present, it is unclear which process resulted in the increased rapsyn-GFP signal. One might speculate that the presence of the myosin Va tail fragment either led to an augmented biosynthesis or to a diminished turnover of the synapse marker. As to the formation of rapsyn-GFP clusters, these were reminiscent of vesicle clusters observed in skin melanocytes [32] and neuroendocrine PC12 cells [35] upon similar treatments, suggesting a related basic mechanism behind such cluster formation. Wu et al. proposed a model in which myosin Va acts through a capture mechanism whereby it retains vesicles travelling along microtubules in the F-actin-rich cell cortex [32]. Although the composition of the postsynaptic apparatus of the NMJ exhibits much more complexity than the C2C12 myotube system it is also characterised by the presence of nuclei [2] in few micrometers distance from the membrane as well as submembranous F-actin [49], which is structured by components of the dystrophin-associated protein complex and the dystrophin-related protein, utrophin [49]. Given the particularly well developed F-actin cytoskeleton underneath the NMJ and the presence of myosin Va on vesicles positive for AChR and rapsyn-GFP, myosin Va might play a role in capturing NMJ-related vesicles to facilitate their incorporation into the postsynaptic membrane, and thereby act in a similar manner as in skin and neuroendocrine cells.

If myosin Va was important for the trafficking of NMJ components, it should be present in their vicinity. To address this issue, we performed biochemical pull-down, *in situ* immunofluorescence and *in vivo* microscopy assays (Figs. 3 and 4). A combination of different biochemical approaches, where either surface-exposed and internalised (pool 1) or the total AChR populations (pool 2) were precipitated, showed an enrichment in tibialis anterior and triceps muscles of myosin Va only in pool 1 but not in pool 2 of AChRs. This result argues for a direct or indirect association of myosin Va with surface-exposed and/or internalised AChRs. The presence of endogenous myosin Va at the NMJ was further substantiated by *in situ* immunofluorescence labelling of the motor protein, since this showed up in numerous punctate structures and aggregates in the close vicinity of the AChR labelling. Furthermore, we found that also a fluorescently marked full length myosin Va and a cargo binding tail fragment of myosin Va exhibited very similar distributions as endogenous myosin Va in live mouse muscle. Finally, *in vivo* co-localisation studies with co-expressed rapsyn-GFP and endocytosed BGT-AF647 staining showed a partial overlap of these signals. Together, these results demonstrate the enrichment in punctate and cluster-like structures carrying myosin Va in close vicinity to the NMJ. Further, they strongly suggest that myosin Va interacts with AChRs, and in fact mainly with those receptors which are either on the cell surface or internalised, thus according to literature using endocytic or recycling pathways [21], [22].

For a number of *in vivo* experiments we made use of a muscle-specific transfection protocol that allows the expression of heterologous proteins and siRNAs in hind limb muscles of adult wildtype mice [38]–[40]. As shown by our previous experiments on second messenger handling and protein transport in live contracting mouse muscle [39], [50], [51], these preparations show no overt defects in innervation. We have now substantiated this notion by performing immunohistochemical stainings of neurofilament M and force measurements in muscles transfected with FLAG-MCLT or silMyoVa (Fig. S3). First, these experiments showed that each postsynapse exhibited a corresponding presynaptic terminal. Second, we found that the maximal force produced by silMyoVa transfected hindlimb muscles was very similar to untransfected control muscles both, when stimulated via the sciatic nerve as well as through an electrode applied directly on the muscle. Interestingly, muscle force was slightly (but not significantly) weaker in silMyoVa transfected muscles. Together, these results demonstrate on the one hand that our muscle transfection protocol *per se* did not lead to any detectable interference with the functional integrity of NMJs and muscle; on the other hand, the morphological changes on NMJs as induced by the functional impairment of myosin Va may have led to small effects on synaptic transmission resulting in slightly weaker contractile responses. Another interesting phenomenon observed during these force measurements was that fatigue upon muscle electrode stimulation was reached much faster in control than in silMyoVa transfected animals. This effect might be explained by a compensatory mechanism of the muscle fibres to counterbalance the muscle weakness in the absence of functional myosin Va. Alternatively, there may exist additional roles of myosin Va within the muscle fibre which led to an increased fatigue resistance upon impairment of the motor protein. Such role needs to be analysed in more detail in future investigations. Using the *in vivo* transfection approach we show evidence that blockage of myosin Va function in adult wildtype mice leads to changes in the morphology of NMJs in a way that they resemble NMJs in *dilute* mice (Fig. 5 and Table 3). Correlative *in vivo* measurements of fibre diameters allowed ruling out an indirect effect of the fibre diameter on the NMJ area.

Further information is also provided on the distribution and size of differentially labeled pools of AChRs (Fig. 6). First, we confirm earlier findings from ectopic rat NMJs regarding the distribution of newly and previously added AChRs [52]; i.e. we found that, albeit in control muscles new and old receptors largely overlapped, BGT stainings of newly arriving or old receptors showed their highest densities at the rim or the centre of NMJs, respectively [see also 53]. This suggests that AChRs exhibit considerable lateral mobility within the NMJ [see also 42] and that the sites of initial addition and subsequent dwelling of AChRs might differ. Second, in the case of silMyoVa transfection, fluorescence signals of new and old receptors were located in largely separate regions. This could either indicate a distinct mobility of AChRs within the NMJ in the absence of functional myosin Va or that under this condition at least a part of the clustered old AChR staining was not within the postsynaptic membrane but only in close proximity to it.

Our biochemical and fluorescence localisation data suggest, that myosin Va predominantly interacted with endocytosed or recycling AChRs (Figs. 3 and 4). If that was the case, one would expect that impairment of myosin Va function should alter or, most likely, inhibit such mechanism. Along that line, we reasoned that inhibition of endocytosis should yield rather larger than smaller NMJs, thus contradicting our findings. If, however, myosin Va facilitated recycling of endocytosed AChRs towards the NMJ, blockage of the motor protein would be expected to diminish NMJ size; furthermore, if there were multiple rounds of AChR recycling one would anticipate that the loss of AChRs should aggravate over time. This expectation was precisely matched in a double-labelling experiment, in which AChRs were subsequently labeled with two distinctly coloured BGT species at different time points in the presence or absence of silMyoVa (Fig. 6). This corroborates earlier findings of Bruneau and colleagues who suggested an important role of AChR recycling in the course of NMJ plasticity [21], [22].

AChR expression in muscle changes during development: While embryonic gamma-subunit-containing AChRs are required for the correct positioning of synaptic contacts during ontogenesis [54], they are replaced postnatally by adult epsilon-subunit-containing AChRs. The latter guarantee a constant, high AChR density and a stable structural organisation of the postsynaptic apparatus [55]. One aspect of this transition is that AChR expression becomes stabilised and restricted to subsynaptic regions while extrajunctional expression is largely reduced [13]. Given that junctional AChRs in adult muscle undergo periodical recycling as recently described [21], it is tempting to speculate in the light of our data that myosin Va mediated AChR recycling is manifested only after the supply of extrajunctional AChRs (predominantly gamma-subunit-containing AChRs) has been depleted. This could explain why impaired recycling of junctional receptors leads to movement disorders in *dilute* mice at a relatively late state, i.e. in the second postnatal week. A scenario in which adult AChRs get recycled would be similar to the situation for a number of other cell surface receptors, including the AMPA receptor in the central nervous system [56], [57]. In this context, a recent work describing the important role of myosin Va in the recycling of the AMPA receptor during synaptic plasticity of hippocampal neurons [34] underscores the probability of an analog function of myosin Va also for the recycling of AChRs at the NMJ.

In conclusion, we have shown *in vitro* and *in vivo* data supporting a crucial function of myosin Va in muscular postsynaptic plasticity. Our data are consistent with a model where myosin Va is present on vesicles containing previously surface-exposed proteinaceous components of the NMJ, such as AChR, and where inhibition of the myosin motor leads to a decrease in NMJ size due to a block of recycling of these components. The correlation we observed between postnatal aetiopathology and the fragmentation and size reduction of NMJs in *dilute* mice lacking functional myosin Va allows to speculate whether the block of recycling NMJ components is partially causative for the severe, lethal seizures in these animals.

## Materials and Methods

### Expression plasmids and chemicals

Experiments used the following constructs: rapsyn-GFP expression vector was from Dr. Cohen (Harvard Medical School, Boston, MA). MCST-YFP and MCST-GFP, expressed in pEYFP-C1 and pEGFP-C1 (BD Bioscience Clontech), respectively, were from Dr. Gerdes (University of Bergen, Norway). FLAG-MCLT and brain full length myosin Va-GFP were as described [32], [58]. SilMyoVa construct used pSilencer™ puro 2.1-U6 (Ambion) and the target sequence: GTTTGCCAGGGTTTGGATC; shuffled control: GTCGGCTAGCTAATGCATC. Antibodies: anti-neurofilament M (Chemicon), anti-synaptophysin (SySy); anti-GM130, anti-AChRalpha and anti-calreticulin (BD Bioscience); anti-myosin Va (LF-18, Sigma), anti-myosin Va (ham5) [32], anti-beta1-adrenergic receptor (Affinity BioReagents); goat-anti-mouse-Cy3, goat-anti-mouse-AF488 and goat-anti-rabbit-AF488 (Invitrogen); goat-anti-mouse-HRP (Daco). BGT-biotinXX, BGT-AF647 and BGT-AF555 (Invitrogen). Draq5 was from Biostatus Limited. NeutrAvidin Agarose and ECL solution were from ThermoScientific and Amersham, respectively. All other chemicals were from Sigma, unless stated differently, and of highest available grade.

### Animals and Transfection

C57BL/6J, C57BL/10J mice and DLS/LeJ (P7–21) mice were used. Animals were from Charles River and then maintained in the local animal facility. Use and care of animals was as approved by German authorities and according to national law (TierSchG §§7). An intraperitoneal (i.p.) injection of Rompun (Bayer) and Zoletil 100 (Laboratoires Virbac) was used for anaesthesia. Transfection was carried out as previously described [38], [39].

### Myoblast culture and immunostaining

C2C12 mouse myoblasts (ATCC) were cultured in DMEM/10% FBS (GIBCO) until they reached 70% confluency. Then, cells were transfected using Lipofectamine2000 (Invitrogen) according to manufacturer's instructions and 1 day later the medium was replaced with DMEM/2% horse serum (GIBCO) for differentiation. Upon 7 days of differentiation, immunofluorescence staining was performed as previously described [35].

### Whole mount muscle staining

Muscles were treated as described [54] with collagenase (Typ 1A, Sigma) for 15 minutes and fixed in 1% formaldehyde for 60 minutes. After incubation in 0.1 M glycin over night muscles were permeabilised in 1% triton/PBS for 8 hours. Primary antibody was diluted in 2% BSA/PBS and incubated for 1 or 2 nights at 4°C. After washing, secondary antibody and BGT-AF647 were added and incubated over night at 4°C. Imaging was done after washing in 2% BSA/PBS and PBS.

### Cryopreservation, sectioning and staining of muscle

As previously described [54] muscles were extracted, washed and fixed over night at 4°C in 4% wt/vol paraformaldehyde/PBS, dehydrated in sucrose solution (10% wt/vol sucrose in distilled water) for 6 h at 4°C, embedded in tissue freezing medium (Labonord), frozen in liquid nitrogen and then stored at −80°C. Longitudinal sections were made at −20°C using a Leica CM1900 cryostat and placed on electrostatic slides (Labonord). Sections were quenched in 50 mM NH_4_Cl, permeabilised with 0.1% TritonX-100, blocked with 10% FBS/PBS and then incubated with 0.2 pM BGT-AF647/ 0.07 pM BGT-AF555/ 10% FBS/PBS. Slices were washed and embedded in Mowiol (Calbiochem).

### Microscopy

All images were taken with a DMRE TCS SP2 confocal microscope equipped with Leica Confocal Software 2.61, a KrAr laser (488 nm, 514 nm), a diode-pumped laser (561 nm), a HeNe laser (633 nm), a 63×/1.4NA HCX PL APO CS oil immersion objective for fixed samples, a 20×/0.7NA HC PL APO CS IMM/CORR UV or a 63×/1.2NA HCX PL APO CS W CORR objective (immersion medium, Visc-Ophtal gel, Winzer-Pharma) (all Leica Microsystems) for *in vivo* observation. ***Fixed samples:*** GFP (excitation 488 nm), Cy3 (excitation 561 nm) and Draq5 signals (excitation 633 nm) of C2C12 cells were acquired using 500–540 nm, 600–630 nm and 650–750 nm bandpass settings, respectively, in the SP2 unit. 3D image stacks were taken at 8-bit, and 1024 or 512 pixel resolution with either 1× or 4× zoom, respectively. BGT-AF647 signals from slices were visualised using 633 nm excitation and emission detection from 650–750 nm. Images were taken at 512 pixel resolution and 8- and 12-bit for slices from wildtype and DLS/LeJ mice, respectively. ***In vivo:*** 10 days after transfection animals were anaesthetised and transfected muscles exposed for microscopy. Fibers were imaged up to a depth of about 400 µm as indicated. Images were taken at 8-bit, 512 pixel resolution, 200 Hz scan frequency, 2× line average. To determine NMJ areas, 25 pmol of BGT-AF647 and/or BGT-AF555 were injected intramuscular at least 30 minutes prior to microscopy. GFP (excitation 488 nm, emission 495–535 nm) and AF647 signals (excitation 633 nm, emission 660–750 nm) as well as AF555 (excitation 561 nm, emission 570–620 nm) and AF647 signals (excitation 633 nm, emission 660–750 nm) were imaged simultaneously. In Fig. 4, YFP (excitation 514 nm, emission 550–590 nm) was imaged subsequent to simultaneous acquisition of GFP (excitation 488 nm, emission 490–510 nm) and AF647 (excitation 633 nm, emission 660–750 nm).

### Data analysis

Image analysis employed ImageJ program. ***Analysis of NMJ size on slices (*** ***Fig. 1*** ***):*** Image stacks of BGT-AF647 signals were median filtered (1 pixel kernel) and thresholded from 50–255 (8-bit data) or 600–4095 (12-bit data) greyscale values. Then, areas within these masks were determined for each individual NMJ. ***C2C12 cells (*** ***Fig. 2*** ***):*** For quantification of rapsyn-GFP signals, images were binarised (50–255 greyscale threshold) upon background subtraction. On maximum z-projections, thresholded signals were counted as GFP-positive areas. ***Analysis of NMJ areas and fibre diameters*** **in vivo** ***(*** ***Fig. 5*** ***):*** Upon background subtraction BGT-AF647 signals were median filtered (0.5 pixel kernel), maximum z-projections made and these then thresholded from 50–255. For determining the sizes of NMJs, signals in the threshold masks were measured. For determining fibre diameters, the largest diameter for each fibre corresponding to analysed NMJs was determined in the YFP channel. ***Analysis of NMJ volumes and areas*** **in vivo** ***(*** ***Fig. 6*** ***):*** Upon background subtraction BGT-AF555 and BGT-AF647 signals were median filtered (0.5 pixel kernel) and thresholded from 50–255. For determining the volume of single NMJs, signals in all single images were measured and cumulated. For determining NMJ areas, maximum z-projections of binarised 3D stacks were analysed. ***Analysis of vesicle co-localisation (*** ***Figs. 4*** ***and*** ***7*** ***):*** 3D image stacks were background-subtracted and median filtered (0.5 pixel kernel). MCST-YFP (Fig. 4) or rapsyn-GFP signals (Fig. 7) were binarised (20–255 greyscale threshold) and screened for objects >4 and <20 pixels^2^. Found object outlines were used as masks in corresponding BGT-AF647 or rapsyn-GFP or MCST-YFP image stacks. Objects within these masks above background were counted as co-localising.

### AChR pull down and Western blot

Mice were injected i.p. with BGT-biotinXX or left untreated prior to tissue extraction. Brain and muscle lysates were made using PreCellys24 homogeniser (PeqLab) with lysis buffer (50 mM Tris-Cl (pH 8.0), 150 mM NaCl, 1% NP-40, 10% glycerol, 1 mM EDTA, 1 mM EGTA, 2 mM Na_3_VO_4_, 1 mM NaF, 0.5 mM PMSF, 1 mM DTT, 10 mM glycerol phosphate and Complete Protease Inhibitors (Roche)) and then centrifuged for 10 minutes at 4000× g. Supernatants of lysates from untreated mice were then incubated with BGT-biotinXX. Subsequently, all supernatants were incubated with 200 µl NeutrAvidin beads (over night, 4°C), centrifuged for 1 minute at 500× g, washed 3 times with lysis buffer, centrifuged again for 1 minute at 500× g and eluted by boiling in Laemmli buffer [59]. After a final centrifugation for 1 minute at 500× g, aliquots of the supernatant (affinity precipitates) and of the lysates were subjected to SDS-PAGE and Western blot analysis as previously described [35]. Western blot analysis of C2C12 cells was carried out as described [60].

### Statistics and graphics

All numeric data were handled using Microscoft Excel 2004 for Mac, version 11.5. All data shown in graphs represent mean±s.e.m. unless otherwise stated. Significance was tested using student's t-test, if applicable. Significance levels are indicated in figures and tables (p≤0.05, *; p≤0.01, **). For data compilation, Adobe Photoshop CS2, version 9 and Adobe Illustrator CS2, version 12.0.0 were employed.

## Supporting Information

Figure S1 ***Active directed movement is increasingly compromised in DLS/LeJ mice during postnatal development as seizures become longer and more intense.*** *Dilute* and healthy littermates were filmed in a standard cage (16×23 cm floorage) at different time points after birth (P9–P20, indicated). **A:** Representative trajectories of the unrestrained animals' movements during a time period of 5 minutes. Note, that at P13 and P20 the *dilute* animal is no more able to actively follow the healthy littermates. In particular, at P20, the movement activity of healthy animals is much higher than that of the *dilute* sibling. Also, most of the movements still observed for the *dilute* mouse were rather due to uncontrolled seizures than to coordinated movements (see also Movies S2, S3, S4). Scalebar, 5 cm. **B:** Quantification, mean velocity of all observed animals. Data, mean±s.e.m. (n = 6 and n = 3 for healthy and *dilute* mice, respectively, from 3 different litters). For each animal and time point the recording time was ten minutes. Movements were recorded using the MTrackJ plugin of ImageJ. While healthy animals show increasingly higher average speed as they grow, *dilute* mice do not gain velocity with age. **C:** Quantification, mean number of seizures per minute of all observed animals. Seizures were detected by visual inspection of the original videos taken at 30 frames per second (fps). Data, mean±s.e.m. (P9, n = 5; P11, n = 6; P13, n = 4; P15, n = 5; P17, n = 3; P20, n = 2. All n-values, animals). **D:** Histograms of the duration of seizures in *dilute* mice as a function of age. Video data sets as in C. Note, that with increasing age seizures become longer. At P20, they often extend over more than a minute and involve the whole body musculature, while at earlier time points contractures are much shorter and are mainly restricted to the limb muscles.
(0.38 MB TIF)

Figure S2 ***GM130 staining is located around the nuclei of C2C12 myotubes.*** C2C12 cells were differentiated for seven days, fixed and stained with an antibody against the cis-Golgi marker GM130 and Draq5 to reveal nuclei. Then, confocal microscopy was performed. Images show fluorescence signals for GM130, Draq5 or the overlay of both signals, as indicated. In the overlay images GM130 and Draq5 signals appear red and blue, respectively. Scale bars, 50 µm. **A:** Field overview showing a single optical slice of some polynucleated myotubes and some mononucleated, less differentiated cells. **B and C:** Blow-up of the lower right myotube in A. B, single optical slice. C, maximum z-projection of 10 confocal slices taken at an interslice distance of 1 µm.
(1.57 MB TIF)

Figure S3 ***Muscles transfected*** **in vivo** ***with FLAG-MCLT or silMyoVa are innervated and contract similar to control muscles.*** TA (A) or EDL (B–F) muscles of adult wildtype mice were either transfected with FLAG-MCLT (A) or silMyoVa (B–F), or left untransfected (control, B and D–F). Ten days later, muscles were fixed and stained using anti-neurofilament M antibody and BGT-AF647 (A) or tested with muscle force measurements (B–F). **A:** Maximum z-projection of 61 slices taken at an interslice distance of 1 µm. Overlay image, BGT-AF647 (red), neurofilament M (green), overlay of both signals (yellow). Scale bar, 50 µm. **B–F:** Isometric force recordings of EDL muscles were performed stimulating either the sciatic nerve or the EDL muscle directly with nerve or muscle electrodes, respectively (indicated). For the recordings, the anaesthetised animals were mounted on a custom-made solid support equipped with micromanipulators to carry electrodes and force transducer. Custom silver (nerve electrode) and platinum electrodes (muscle electrode) were used with a Master-8-cp stimulator (A.M.P.I.). The nerve electrode was inserted next to the sciatic nerve, the muscle electrode was placed on top of the muscle and both spots were supplied with physiological solution. Force recordings were made using an MLT050/A isometric force transducer connected to a PowerLab 8/30 recorder (both ADInstruments) with a recording frequency of 2 kHz. Stimulation was performed using 5 ms pulses in trains of 500 ms duration with a frequency of 100 Hz every 4 seconds until half maximal force was reached (fatigue). Then, recovery from fatigue was verified by single 500 ms trains every minute (recovery). **B and C:** Representative force traces of control muscles (B) or muscles transfected with silMyoVa (C). The pause between nerve and muscle stimulation recordings was in each case 10 minutes long. **D:** Quantification of the average maximum isometric force produced by muscles upon nerve or muscle stimulation (indicated). Data, mean±s.e.m. (n = 6 mice). Note, that maximum forces are similar in all conditions. Force production upon nerve stimulation in silMyoVa transfected muscles is slightly, but not significantly, reduced. **E:** Quantification of the number of tetani needed to reach 50% of fatigue upon nerve or muscle stimulation (indicated). Data, mean±s.e.m. (n = 6 mice). **F:** Relative duration to reach 50% of fatigue upon stimulation of muscle or nerve. Shown are the averaged quotients of muscle divided by nerve stimulation values for each tested muscle. Data, mean±s.e.m. (n = 6 mice). Note, that in general fatigue is reached upon muscle stimulation much slower than upon nerve stimulation. Interestingly, this difference is much more pronounced in silMyoVa as compared to control animals, suggesting that these muscles have undergone some adaptive processes to maintain a certain level of functionality as effect of the NMJ deteriorating action of silMyoVa transfection.
(1.22 MB TIF)

Movie S1 ***Movement of DLS/LeJ mice is normal at P9.*** Video of two healthy (black) and one *dilute* (grey) littermates at P9. The video has a duration of one minute; it plays in real time showing 5 fps (original acquisition rate, 30 fps). Cage dimension, 16×23 cm. The *dilute* animal shows no overt seizures and actively approaches its healthy littermates.
(8.68 MB MOV)

Movie S2 ***DLS/LeJ mice exhibit seizures and have lost the ability to follow healthy littermates at P13.*** Video of two healthy (black) and one *dilute* (grey) littermates at P13. The video has a duration of one minute; it plays in real time showing 5 fps (original acquisition rate, 30 fps). Cage dimension, 16×23 cm. The *dilute* animal shows a number of short periods of seizures which mainly affect the limb muscles. The animal is no more able to actively approach its healthy littermates.
(10.87 MB MOV)

Movie S3 ***DLS/LeJ mice exhibit very severe and long-lasting seizures at P20.*** Video of two healthy (black) and one *dilute* (grey) littermates at P20. The video has a duration of one minute; it plays in real time showing 5 fps (original acquisition rate, 30 fps). Cage dimension, 16×23 cm. The *dilute* animal shows seizures with much prolonged duration, which affect the whole body musculature, including the dorsal muscles. This leads to bending and rounding of the body eventually drawing the whole animal on its back. Then, the intense cramps of the limb muscles become easily visible.
(11.52 MB MOV)

Movie S4 ***Endogenous myosin Va is localised in punctate structures close to the NMJ.*** Peronaeus muscle of wildtype mice (P14) was shortly fixed and stained using ham5 anti-myosin Va antibody (green) and BGT-AF647 (red) and then visualised using confocal microscopy. Movie, rotating 3D projection of 78 optical slices taken at an interslice distance of 500 nm. For better visibility of the different signals, the BGT-AF647 signal is omitted after each full turn.
(1.28 MB MOV)

Movie S5 ***Upon transfection of rapsyn-GFP NMJs maintain their normal shape and rapsyn-GFP, old and new AChRs perfectly co-localise in vivo.*** TA muscles of adult wildtype mice were transfected with rapsyn-GFP (green) and simultaneously injected with BGT-AF647 (‘old receptors’, blue). Ten days later, muscles were injected with BGT-AF555 (‘new receptors’, red). Then, fluorescence signals were monitored with confocal *in vivo* microscopy. The movie depicts a rotating 3D projection of 33 optical slices taken at an interslice distance of 800 nm. For better visibility of the different signals, only the BGT-AF647 signal shows up initially, then the BGT-AF555 signal is shown on top after the first full turn and finally the rapsyn-GFP signal is added, as indicated in the lower left corner of the movie. The depicted frame has an actual height of 81 µm. Note, that all three fluorescence signals nicely overlap and exhibit comparable extensions and intensities. Furthermore, the overall morphology of the NMJ is richly branched but contiguous.
(1.68 MB MOV)

Movie S6 ***Upon co-transfection of rapsyn-GFP and silMyoVa NMJs are disrupted and rapsyn-GFP widely co-localises with new AChRs in intracellular punctate structures in vivo.*** TA muscles of adult wildtype mice were co-transfected with rapsyn-GFP (green) and silMyoVa and simultaneously injected with BGT-AF647 (‘old receptors’, blue). Ten days later, muscles were injected with BGT-AF555 (‘new receptors’, red). Then, fluorescence signals were monitored with confocal *in vivo* microscopy. The movie depicts a rotating 3D projection of 53 optical slices taken at an interslice distance of 1 µm. For better visibility of the different signals, only the BGT-AF647 signal shows up initially, then the BGT-AF555 signal is shown on top after the first full turn and finally the rapsyn-GFP signal is added, as indicated in the lower left corner of the movie. The depicted frame has an actual height of 81 µm. Note, that only little ‘old receptors’ remained. These hardly overlap with ‘new receptors’ and rapsyn-GFP. Conversely, rapsyn-GFP and ‘new receptors’ extensively co-localise in numerous, intracellular punctate structures and at the rim of the putative NMJ remainder.
(2.54 MB MOV)
