# Supplementary figures and images for: Role of Myosin Va in the Plasticity of the Vertebrate Neuromuscular Junction In Vivo
Source: PLoS One. 2008 Dec 5;3(12):e3871. doi: 10.1371/journal.pone.0003871 (PMC2587709; doi:10.1371/journal.pone.0003871)

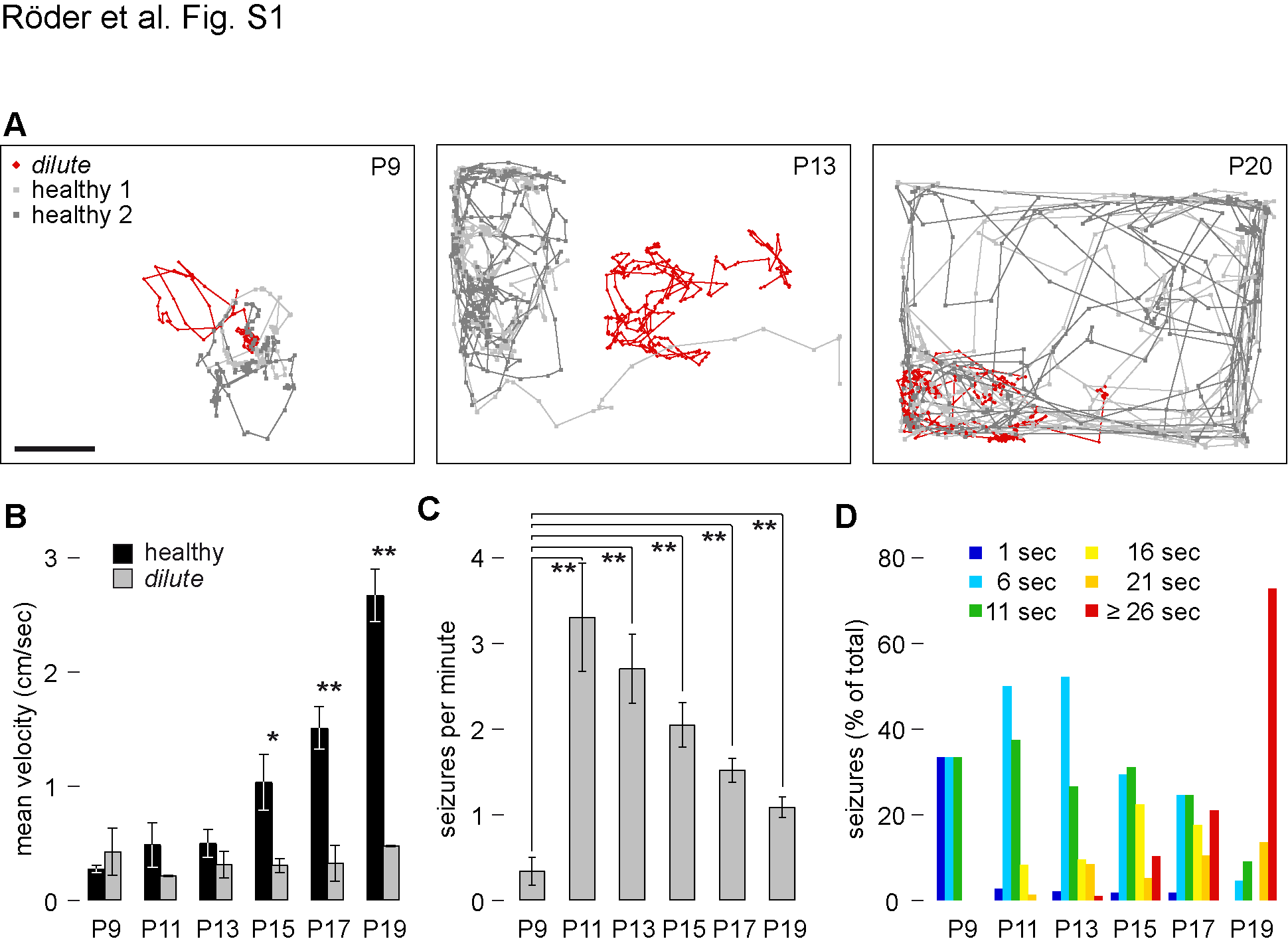

Supplement: Figure S1 — Active directed movement is increasingly compromised in DLS/LeJ mice during postnatal development as seizures become longer and more intense. Dilute and healthy littermates were filmed in a standard cage (16×23 cm floorage) at different time points after birth (P9–P20, indicated). A: Representative trajectories of the unrestrained animals' movements during a time period of 5 minutes. Note, that at P13 and P20 the dilute animal is no more able to actively follow the healthy littermates. In particular, at P20, the movement activity of healthy animals is much higher than that of the dilute sibling. Also, most of the movements still observed for the dilute mouse were rather due to uncontrolled seizures than to coordinated movements (see also Movies S2, S3, S4). Scalebar, 5 cm. B: Quantification, mean velocity of all observed animals. Data, mean±s.e.m. (n = 6 and n = 3 for healthy and dilute mice, respectively, from 3 different litters). For each animal and time point the recording time was ten minutes. Movements were recorded using the MTrackJ plugin of ImageJ. While healthy animals show increasingly higher average speed as they grow, dilute mice do not gain velocity with age. C: Quantification, mean number of seizures per minute of all observed animals. Seizures were detected by visual inspection of the original videos taken at 30 frames per second (fps). Data, mean±s.e.m. (P9, n = 5; P11, n = 6; P13, n = 4; P15, n = 5; P17, n = 3; P20, n = 2. All n-values, animals). D: Histograms of the duration of seizures in dilute mice as a function of age. Video data sets as in C. Note, that with increasing age seizures become longer. At P20, they often extend over more than a minute and involve the whole body musculature, while at earlier time points contractures are much shorter and are mainly restricted to the limb muscles. (0.38 MB TIF) [file pone.0003871.s001.tif]

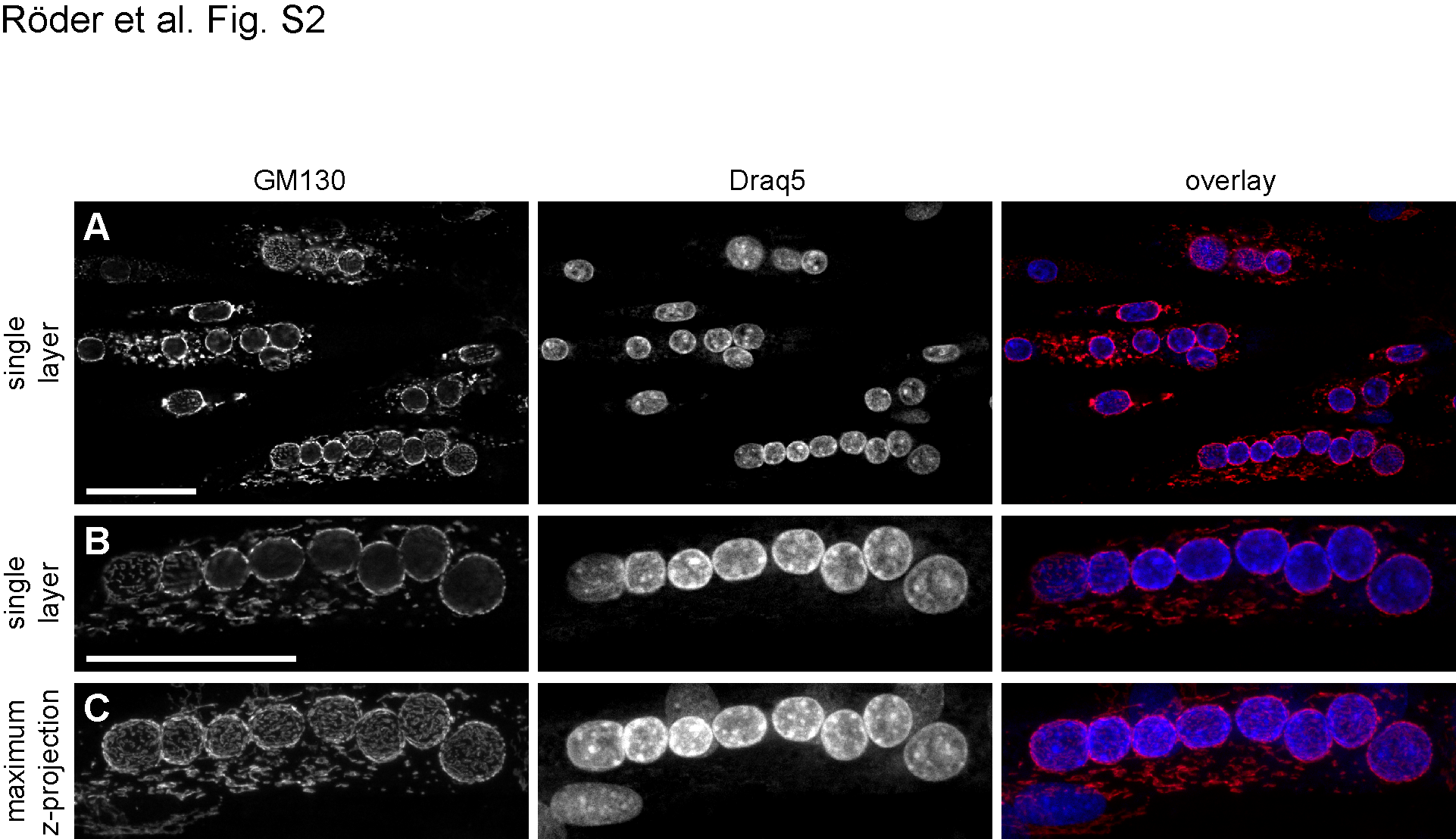

Supplement: Figure S2 — GM130 staining is located around the nuclei of C2C12 myotubes. C2C12 cells were differentiated for seven days, fixed and stained with an antibody against the cis-Golgi marker GM130 and Draq5 to reveal nuclei. Then, confocal microscopy was performed. Images show fluorescence signals for GM130, Draq5 or the overlay of both signals, as indicated. In the overlay images GM130 and Draq5 signals appear red and blue, respectively. Scale bars, 50 µm. A: Field overview showing a single optical slice of some polynucleated myotubes and some mononucleated, less differentiated cells. B and C: Blow-up of the lower right myotube in A. B, single optical slice. C, maximum z-projection of 10 confocal slices taken at an interslice distance of 1 µm. (1.57 MB TIF) [file pone.0003871.s002.tif]

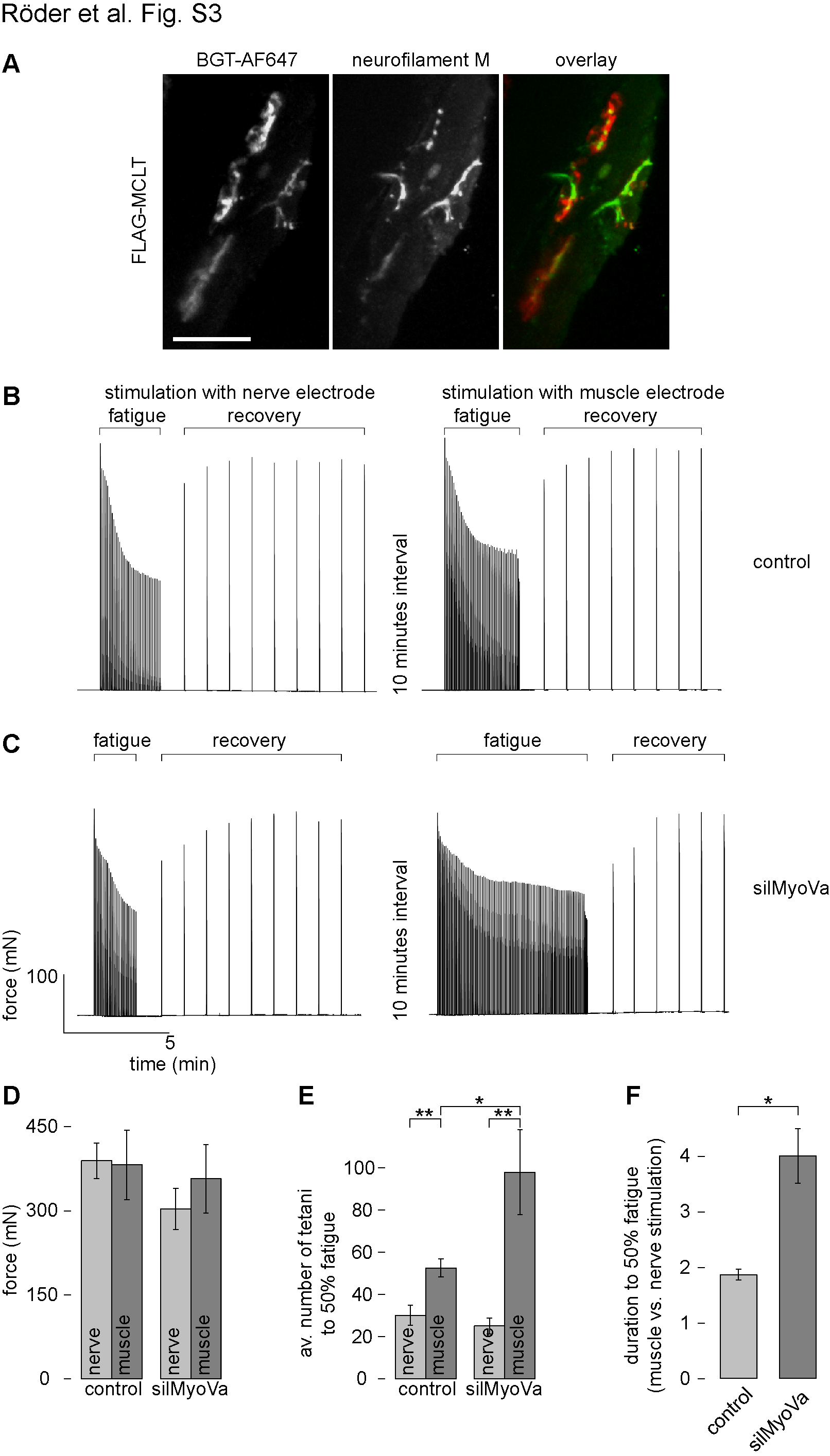

Supplement: Figure S3 — Muscles transfected in vivo with FLAG-MCLT or silMyoVa are innervated and contract similar to control muscles. TA (A) or EDL (B–F) muscles of adult wildtype mice were either transfected with FLAG-MCLT (A) or silMyoVa (B–F), or left untransfected (control, B and D–F). Ten days later, muscles were fixed and stained using anti-neurofilament M antibody and BGT-AF647 (A) or tested with muscle force measurements (B–F). A: Maximum z-projection of 61 slices taken at an interslice distance of 1 µm. Overlay image, BGT-AF647 (red), neurofilament M (green), overlay of both signals (yellow). Scale bar, 50 µm. B–F: Isometric force recordings of EDL muscles were performed stimulating either the sciatic nerve or the EDL muscle directly with nerve or muscle electrodes, respectively (indicated). For the recordings, the anaesthetised animals were mounted on a custom-made solid support equipped with micromanipulators to carry electrodes and force transducer. Custom silver (nerve electrode) and platinum electrodes (muscle electrode) were used with a Master-8-cp stimulator (A.M.P.I.). The nerve electrode was inserted next to the sciatic nerve, the muscle electrode was placed on top of the muscle and both spots were supplied with physiological solution. Force recordings were made using an MLT050/A isometric force transducer connected to a PowerLab 8/30 recorder (both ADInstruments) with a recording frequency of 2 kHz. Stimulation was performed using 5 ms pulses in trains of 500 ms duration with a frequency of 100 Hz every 4 seconds until half maximal force was reached (fatigue). Then, recovery from fatigue was verified by single 500 ms trains every minute (recovery). B and C: Representative force traces of control muscles (B) or muscles transfected with silMyoVa (C). The pause between nerve and muscle stimulation recordings was in each case 10 minutes long. D: Quantification of the average maximum isometric force produced by muscles upon nerve or muscle stimulation (indicated). Data, mean [file pone.0003871.s003.tif]
